# Short-Time Changes in Coronary Artery Plaques Assessed by Follow-Up Coronary CT Angiography—Characteristics and Impact on Patient Management

**DOI:** 10.3389/fcvm.2021.691665

**Published:** 2021-08-09

**Authors:** Hanna Maria Görich, Sebastian J. Buss, Mostafa Emami, Sebastian Seitz, Dirk Lossnitzer, Philipp Fortner, Stefan Baumann, Matthias Brado, Friedemann Gückel, Roman Sokiranski, André Sommer, Johannes Görich, Florian Andre

**Affiliations:** ^1^Radiology Center Sinsheim-Eberbach-Erbach-Walldorf-Heidelberg, Heidelberg, Germany; ^2^Department of Diagnostic and Interventional Radiology and Nuclear Medicine, University Medical Center Hamburg-Eppendorf, Hamburg, Germany; ^3^First Department of Medicine – Cardiology, University Medical Center Mannheim, Mannheim, Germany; ^4^Department of Cardiology, Angiology and Pneumology, University of Heidelberg, Heidelberg, Germany

**Keywords:** coronary CT angiography, coronary artery plaques, dual-source CT, coronary artery diseae, follow-up examinations, short-time change detection

## Abstract

**Background:** Coronary artery disease (CAD) shows a chronic but heterogeneous clinical course. Coronary CT angiography (CTA) allows for the visualization of the entire coronary tree and the detection of early stages of CAD. The aim of this study was to assess short-time changes in non-calcified and mixed plaques and their clinical impact using coronary CTA in a real-world setting.

**Methods:** Between 11/2014 and 07/2019, 6,701 patients had a coronary CTA with a third-generation dual-source CT, of whom 77 patients (57 males, 63.8 ± 10.8 years) with a chronic CAD received clinically indicated follow-up CTA. Non-calcified and mixed plaques were analyzed in 1,211 coronary segments. Patients were divided into groups: stable, progressive, or regressive plaques.

**Results:** Within the follow-up period of 22.3 ± 10.4 months, 44 patients (58%) showed stable plaques, 27 (36%) showed progression, 5 (7%) showed regression. One patient was excluded due to an undetermined CAD course showing both, progressive and regressive plaques. Age did not differ significantly between groups. Patients with plaque regression were predominantly female (80 vs. 20%), whereas patients showing progression were mainly male (85 vs. 15%; *p* < 0.01 for both). Regression was only observed in patients with mild CAD or one-vessel disease. The follow-up CTA led to changes in patient management in the majority of subjects (*n* = 50; 66%).

**Conclusions:** Changes in coronary artery plaques can be observed within a short period resulting in an adjustment of the clinical management in the majority of CAD patients. Follow-up coronary CTA renders the non-invasive assessment of plaque development possible and allows for an individualized diagnostics and therapy optimization.

## Introduction

Coronary artery disease (CAD) is the most common cause of death worldwide having a considerable socio-economic impact (1). Atherosclerosis of the coronary arteries begins in childhood and continues to develop for decades until it may eventually become clinically overt (2). CAD is a dynamic process often showing a stable course over long periods, however, it can become unstable at any time. Of note, by far most of all cardiovascular events are due to the rupture of plaques of non-stenotic vascular wall alterations with subsequent multifactorial coagulation cascade initiation and consecutive vascular occlusion (3–5). Hence, CAD has to be regarded rather as a chronic than as a stable disease requiring follow-up care even in asymptomatic patients as highlighted in recent guidelines (6). Coronary CT angiography (CTA) has recently been established as a highly sensitive imaging modality for the detection of CAD. Compared to invasive coronary angiography, it not only determines the severity of stenosis but also visualizes the plaque composition and, thus, can differentiate between non-calcified, calcified, and mixed plaques. Due to the slow course of atherosclerotic plaque progression and cumbersome plaque characterization in non-invasive techniques, evaluation of plaque morphology is rarely performed asides from studies in clinical routine. Correspondingly, catheter-based procedures such as intravascular ultrasound (IVUS) and optical coherence tomography (OCT) with a high spatial resolution are not conducted routinely because of their invasive nature. Of note, they render only the evaluation of distinct lesions possible. In contrast, coronary CTA allows for an easy, non-invasive assessment of the entire coronary tree. Besides the detection of critical lesions, non-culprit plaques can be assessed thoroughly before becoming clinically relevant. Modern CT scanner technology and advanced image acquisition and reconstruction techniques offer high temporal and spatial resolution in combination with low radiation exposure leading to a better understanding of coronary plaque composition and vulnerability (5). However, data on plaque development in individual patients were predominantly obtained in clinical studies and, thus, data on its clinical significance in real-world populations is scarce. We hypothesized that radiologically detectable changes in plaque morphology may occur within a relatively short period in patients with chronic CAD, potentially influencing the further clinical approach. In this study, we assessed the dynamic alterations of non-calcified and mixed coronary artery plaques using a dual-source CT (DSCT) scanner of the third generation and their impact on patient management in a real-world population.

## Materials and Methods

### Study Population

Between 11/2014 and 07/2019, 6,701 coronary CTA examinations were performed in stable outpatients at a radiology center. All patients, who received another coronary CTA at least 6 months after the initial examination, were identified and included in the study (*n* = 77). The follow-up examination was indicated by the referring physician and, thus, no dedicated study protocol was applied. Demographic data and cardiovascular risk factors were taken at the initial presentation.

### Patient Preparation

Before coronary CTA, up to 20 mg metoprolol tartrate (Lopressor, Recordati Pharma, Ulm, Germany) was administered intravenously in patients without contraindications to achieve a heart rate of <65 /min. Glycerol trinitrate (up to 0.8 mg, Nitrolingual, Pohl-Boskamp, Hohenlockstedt, Germany) was applied sublingually in all patients before the scan to optimize the coronary artery visualization. Since image acquisition was performed in inspiration breath-hold, patients were trained in breathing maneuvers.

### Data Acquisition and Post-processing

Coronary CTA scans were performed on a DSCT system of the third generation (SOMATOM Force, Siemens Healthcare, Erlangen, Germany) with integrated circuit detectors (Stellar Infinity). Calcium scoring was performed prior to contrast agent administration. A contrast agent bolus of 60–80 ml Iomeprol (Imeron 400, Bracco Imaging, Konstanz, Germany) depending on the body weight was injected continuously and followed by a flush of 30 ml saline solution with an injection rate of 5 ml/s. The region of interest for bolus tracking was placed in the aortic root and image acquisition was started after a predefined threshold of 100 Hounsfield units (HU) was reached. Calcium scoring results were used for the optimization of subsequent CTA protocol. Routinely, a helical acquisition protocol with automatic pitch mode and automated attenuation-based tube potential and current selection (CARE Dose4D, Siemens Healthcare, Erlangen, Germany) was applied. Collimation was set to 96 × 0.6 mm and a slice acquisition of 192 × 0.6 mm by means of a z-flying focus was used. A fixed ECG pulsing window of 40–75% of the RR interval with a reduction to 4% of the tube current outside that window was applied. Hence, multi-phase reconstructions were available providing good image quality even in patients with irregular heart rhythm or high plaque burden. In some patients with regular heart rhythm and negligible coronary calcium burden, a turbo high-pitch spiral mode (pitch 3.2, table feed 737 mm/s) with automatic heart phase selection was applied. Datasets were reconstructed using the Advanced Modeled Iterative Reconstruction Algorithm (ADMIRE, strength level 3) with a medium soft tissue kernel (Bv36 or Bv40). Image analyses were performed using dedicated software (syngo.via, Siemens Healthcare, Erlangen, Germany).

### Assessment of Coronary CTA Examinations

Coronary CTA examinations were prospectively assessed in a consensus reading by a cardiologist and a radiologist, both having an experience >4000 CTA examinations, applying the current guidelines of the Society of Cardiovascular Computed Tomography for interpretation and reporting of coronary CTA (7). Coronary arteries were visually assessed on axial images as well as on curved and straightened multiplanar reformations. If a stenosis was detected, its severity was measured using the vendor's dedicated software comparing the diameter of the stenotic part to the diameter of the adjacent non-stenotic segments. Coronary arteries were segmentally graded as normal or with minimal, mild, moderate, or severe stenosis, or occlusion (7, 8). In addition, the quality of the plaques (non-calcified, mixed, calcified) and high-risk features (remodeling, spotty calcification, napkin ring, fibrous cap) were assessed (7–10). For the determination of non-calcified and mixed plaque alterations, the reports and the images of both examinations were compared especially giving attention to changes in size and diameter stenosis of plaques, the concomitance of high-risk features, and the occurrence of new lesions. Calcified plaques, stented lesions, and bypassed coronary arteries were not included in the analysis. The patients were classified in consensus with regard to the global plaque development into three different CAD groups: stable non-calcified and mixed plaques (I), progressive non-calcified and mixed plaques (II), regressive non-calcified and mixed plaques (III). CAD was regarded as stable if no significant changes in plaques occurred. An increase in the length and diameter stenosis leading to a higher severity grading (e.g., mild to moderate) and the occurrence of new lesions resulted in the classification as progressive CAD. Of note, an increase in the calcified plaque proportion was not regarded as progression, if the total plaque size did not change. In the regressive group, lesions, which were present at the initial CTA examination, needed to decrease. If patients showed plaque progression and regression, the case was classified as undetermined CAD course and excluded from further analyses. In total, 1,211 segments of the patients' coronary arteries were analyzed twice and subsequently compared. Coronary calcification was quantified using the Agatston score equivalent. Additionally, a CT Fractional Flow Reserve (CT_FFR_) evaluation was conducted in three selected cases solely for research purposes and the exemplification of plaque development (11, 12). Blinded to clinical data, a subset of 16 cases was reviewed after more than 12 months in order to assess the agreement on the CAD group assignment. All cardiac CT examinations were clinically indicated by the referring physicians and approval for the retrospective data analysis was obtained from the local ethics committee.

### Statistical Analysis

Continuous data are given as mean ± standard deviation and differences were assessed using a Student's *t*-test or an ANOVA. Non-parametric data are given as median and interquartile range and the Kruskal-Wallis test with a *post-hoc* analysis (Conover) was used for the comparison of multiple groups. Categorical variables are presented as numbers and proportions and were assessed using the Chi-squared test. The intra-class correlation coefficient with a two-way model and absolute agreement was utilized for the assessment of the agreement on CAD group assignment. A *p* < 0.05 was regarded as statistically significant. Statistical analyses were conducted using MedCalc Statistical Software version 19 (MedCalc Software, Ostend, Belgium).

## Results

### Study Population

**Figure 1:**
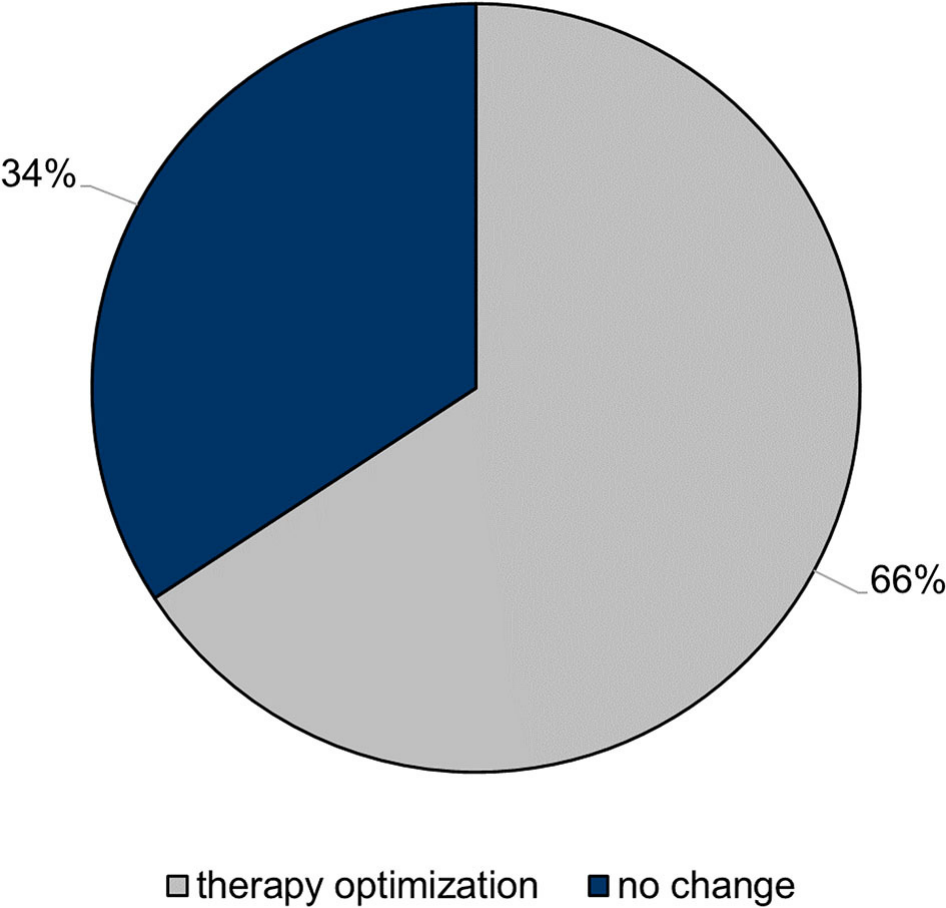
Impact of follow-up coronary CT angiography on patient management. In a relevant proportion of the study population, the therapeutic regimen was adjusted due to the results of the follow-up coronary CT angiography.

The final study population consisted of 76 patients (56 male, 20 female) undergoing two coronary CTA examinations within a mean time period of 22.3 ± 10.4 months. One patient could not be assigned to any group due to an undetermined course of the CAD and had to be excluded (see Figure 1 for details). The mean age was 63.9 ± 10.8 years with no significant difference between men and women (63.8 ± 10.9 vs. 64.3 ± 10.9 years, *p* = n.s.). Patients' characteristics are given in Table 1.

**Table 1:** Characteristics of the patients and CTA results.

	**All**	**Group I**	**Group II**	**Group III**	***p***
	**(*n* = 76)**	**Stable (*n* = 44)**	**Progression (*n* = 27)**	**Regression (*n* = 5)**	
**Demographic data**					
Age	63.9 ± 10.8	63.8 ± 10.3	64.8 ± 12.0	59.4 ± 9.3	n.s.
Sex (m/f)	56/20	32/12	23/4	1/4	<0.01
Body Mass Index (kg/m^2^)	27.0 (24.5–30.1)	28.6 (25.4–30.7)	25.8 (23.7–28.9)	20.3 (20.2–24.3)	<0.05^*^
**Cardiovascular risk factors**					
Arterial hypertension	55 (72%)	31 (71%)	21 (78%)	3 (60%)	n.s.
Hypercholesterolemia	43 (57%)	23 (52%)	15 (56%)	5 (100%)	n.s.
Diabetes mellitus	11 (15%)	8 (18%)	2 (7%)	1 (20%)	n.s.
Smoking	17 (22%)	11 (25%)	5 (19%)	1 (20%)	n.s.
Family History of CAD	39 (51%)	20 (46%)	15 (56%)	4 (80%)	n.s.
**Indication for initial CTA**					
Angina	9 (12%)	3 (7%)	5 (19%)	1 (20%)	n.s.
Dyspnea	6 (8%)	5 (11%)	1 (4%)	0 (0%)	
Atypical symptoms	28 (37%)	15 (34%)	12 (44%)	1 (20%)	
Equivocal stress test	3 (4%)	3 (7%)	0 (0%)	0 (0%)	
Abnormal ECG/echo	8 (11%)	7 (16%)	1 (4%)	0 (0%)	
Increased CAD risk/preOP	19 (25%)	9 (21%)	7 (26%)	3 (60%)	
Other	3 (4%)	2 (5%)	1 (4%)	0 (0%)	
**Indication for follow-up CTA**					
Angina	7 (9%)	4 (9%)	3 (11%)	0 (0%)	n.s.
Dyspnea	7 (9%)	7 (16%)	0 (0%)	0 (0%)	
Atypical Symptoms	44 (58%)	22 (50%)	17 (63%)	5 (100%)	
Equivocal stress test	2 (3%)	0 (0%)	2 (7%)	0 (0%)	
Abnormal ECG/echo	9 (12%)	5 (11%)	4 (15%)	0 (0%)	
Increased CAD risk/preOP	1 (1%)	1 (2%)	0 (0%)	0 (0%)	
Other	6 (8%)	5 (11%)	1 (4%)	0 (0%)	
**CAD**					
Agatston score equivalent	73 (1.0–507.0)	80.6 (0.8–560.5)	79.0 (6.3–268.0)	39.0 (0.8–59.8)	n.s.
Mild CAD	35	24	10	1	<0.001
One-vessel disease	13	4	5	4	
Two-vessel disease	13	4	9	0	
Three-vessel disease	15	12	3	0	
**Imaging values**					
Heart rate (/min) at initial CTA	60.5 (54.0–67.5)	60.0 (52.0–67.5)	61.0 (55.3–66.8)	61.0 (56.8–69.8)	n.s.
Heart rate (/min) at follow-up CTA	58.0 (54.0–64.0)	58.0 (54.0–64.0)	57.0 (53.3–64.3)	59.0 (53.0–62.8)	n.s.
DLP (mGy*cm) at initial CTA	280 (116–493)	420 (162–569)	175 (97–314)	56 (24-119)	<0.001^**^
DLP (mGy^*^cm) at follow-up CTA	495 (343–755)	545 (390–792)	490 (313–715)	277 (147–324)	<0.05^***^

*Mild CAD was defined as the presence of coronary artery plaques resulting in presumably hemodynamically insignificant stenoses <50%. Demographic data and cardiovascular risk factors were obtained at the initial presentation of the patients. Atypical symptoms contain symptoms that are non-specific for CAD as atypical chest pain and syncope as well as the clinical suspicion of a relevant progression of the CAD. Others contain e.g., combined CTA of the coronary arteries and the thoracic aorta. DLP, dose length product; ECG, electrocardiogram; Echo, echocardiography; preOP, preoperatively*.

* *Post-hoc analysis showed significant differences between group I and the other groups (p < 0.05)*.

** *Post-hoc analysis showed significant differences between all groups (p < 0.05)*.

*** *Post-hoc analysis showed significant differences between group III and the other groups (p < 0.05)*.

### Initial CTA Examination

At the initial presentation, 35 patients had a mild form of CAD with plaques resulting in insignificant stenoses (<50%), 13 patients had a one-vessel CAD and 13 patients had a two-vessel CAD. In 15 cases, all coronary arteries showed at least moderate stenoses resulting in a three-vessel CAD. After the initial coronary CTA, 13 patients underwent myocardial revascularization either by percutaneous coronary intervention (*n* = 12) or bypass surgery (*n* = 1). Four patients showed plaques with high-risk features.

### Follow-Up Coronary CTA Examination

At the follow-up CTA examination, 44 patients (58%) showed stable plaques (group I), 27 patients (36%) showed a progression (group II), whereas in 5 patients (7%) the plaques were regressive (group III). The interval between CT scans did not differ significantly between groups (20.1 ± 10.8 vs. 25.7 ± 9.1 vs. 23.7 ± 9.6 months; *p* = n.s.).

**Figure 2:**
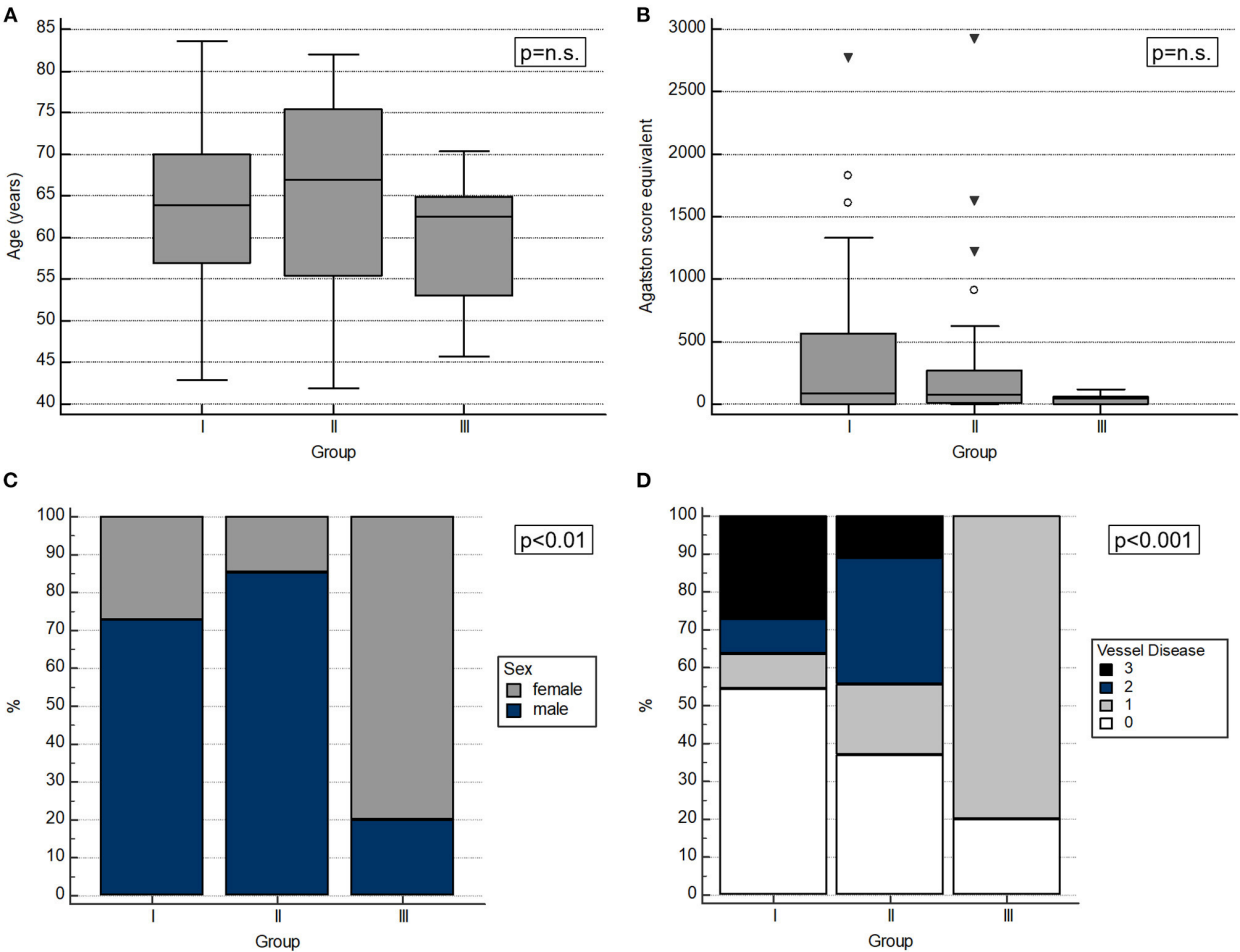
Characteristics of the CAD progression groups. **(A)** Age did not differ significantly between groups. **(B)** The initial Agatston score equivalent showed no significant differences between groups. **(C)** The sex distribution was significantly different between groups with the regression group showing the highest fraction of female patients. **(D)** Patients of the regression group showed solely mild or one-vessel CAD. Of note, the fraction of stenotic coronary arteries differed significantly between groups at the initial presentation.

Differences in the initial age between groups were not significant (63.8 ± 10.3 vs. 64.8 ± 12.0 vs. 59.4 ± 9.3 years; *p* = n.s.). However, the sex distribution varied significantly between groups with group III (regressive plaques) showing the highest and group II (progressive plaques) the lowest proportion of female subjects (27 vs. 15 vs. 80%; *p* < 0.01). Differences in the initial coronary artery calcification between groups did not reach significance [80.6 (0.8–560.5) vs. 79.0 (6.3-268.0) vs. 39.0 (0.8–59.8); *p* = n.s.], whereas the initial fraction of significant stenotic coronary arteries (≥50%) differed significantly between groups with group III (plaque regression) containing solely patients with no significant stenosis or one-vessel CAD (*p* < 0.001). Plaques with high-risk features occurred in 8 patients of whom 7 were in the progression group. The other one underwent a percutaneous coronary intervention in the meantime and showed an overall stable CAD course with the high-risk features being present in a mild stenosis. Data is presented in Figure 2.

**Figure 3:**
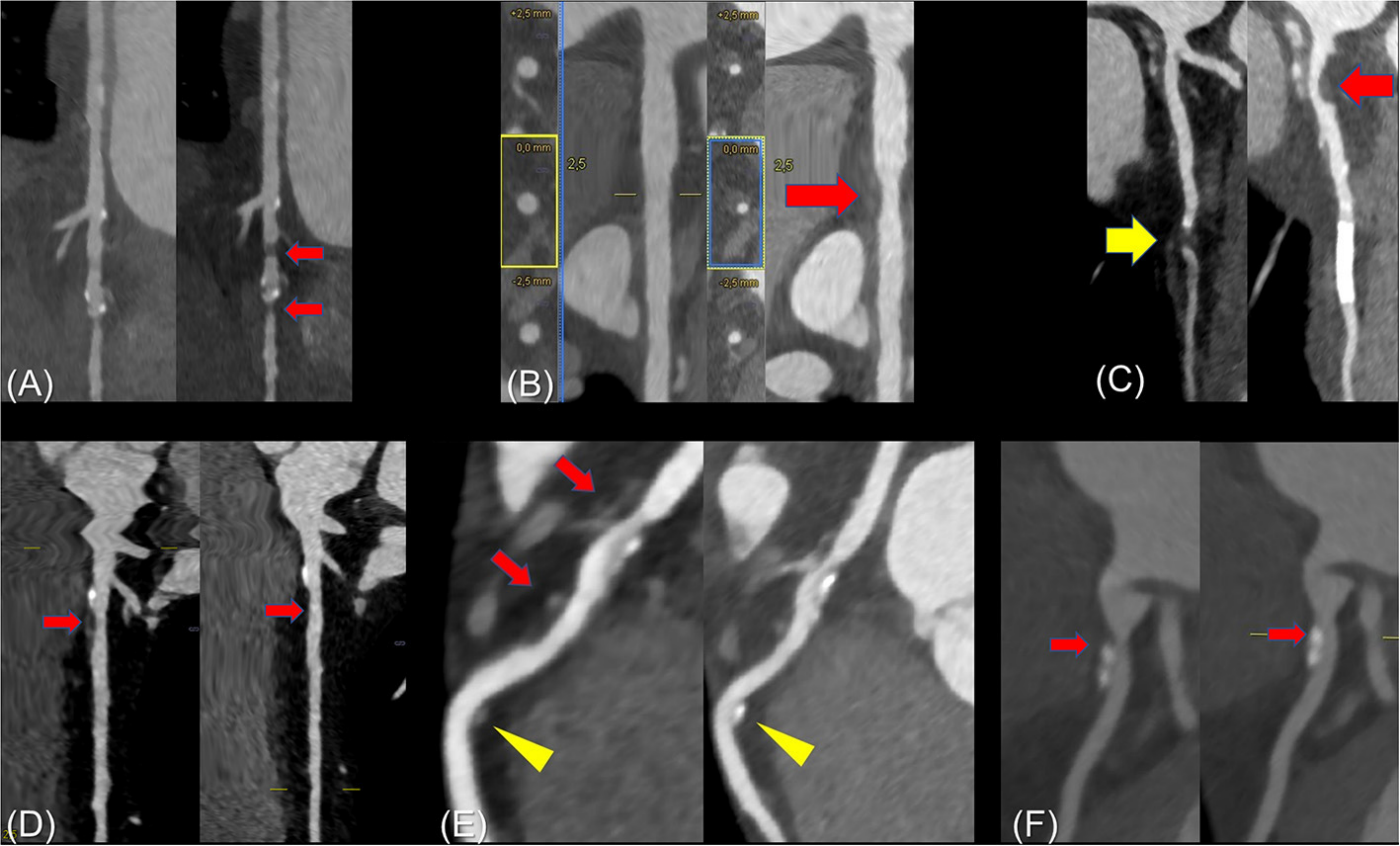
Examples of progressive and regressive plaques. **(A)** Rapidly progressive CAD with the occurrence of two significant plaques in the distal LCX within a period of 14 months. **(B)** Initially, only slight irregularities of the proximal LCX were found. Statin therapy was rejected by the patient. As a malignant thymoma was incidentally found at the combined coronary and thoracic CT examination, the patient underwent radiotherapy, which contained the proximal LCX in the radiation field. In the follow-up CTA examination after 18 months, a non-calcified plaque with a lumen narrowing of ≈50% was found in the LCX with a new inflammatory border in the perivascular fat tissue (arrow). **(C)** The initial coronary CTA showed a short-length closure of the LAD segment 7 (yellow arrow) and slight wall irregularities proximally. The follow-up examination after 44 months demonstrated an open coronary artery stent and a significant progression of atherosclerosis with a new non-calcified plaque in the proximal LAD segment 6 (red arrow). **(D)** Initially, the proximal LAD showed calcifications with an adjacent non-calcified plaque with negative remodeling resulting in a mild stenosis. After 18 months of intensified statin therapy, almost complete regression of the vulnerable plaque with only slight wall irregularities of the vessel wall was observed. Of note, even the soft hem around the proximal calcification was not detectable any longer. **(E)** The predominantly non-calcified plaque (red arrows) with positive remodeling and fibrous cap diminished clearly in the follow-up coronary CTA after 36 months of statin therapy. The distal non-calcified plaque (yellow arrows) almost calcified completely. **(F)** The initial examination showed a dumbbell-like calcification with surrounding soft tissue cover (arrow) of the LAD. After statin therapy for 24 months, an increasing calcification was detectable and the apical non-calcified plaque did not longer appear. The vessel area increased from 2.9 mm^2^ to 3.8 mm^2^.

Image samples of patients showing plaque progression or regression are given in Figure 3 and an example of CT_FFR_-confirmed hemodynamically relevant plaque progression is shown in the Supplementary Materials. Based on the symptoms of the patients, their medication, the available medical reports, and the results of the CTA examinations, recommendations for further diagnostic and therapeutic procedures were given. An adjustment of the medical therapy inclusive of the beginning and increasing of statin therapy or the addition of another lipid-lowering substance was recommended in 10 (13%) and 30 (39%), ischemia or viability assessment in 25 (33%) and 30 (39%), invasive coronary angiography in 13 (17%) and 8 (11%), and other diagnostics (e.g., comprehensive echocardiography) in 3 (4%) and 0 (0%) of the patients at the initial and follow-up examination, respectively. Of note, the second CTA examinations led to the recommendation to adjust the clinical management in 50 subjects (66%).

The intra-class correlation coefficient was 0.93 showing a very good agreement on the CAD group assignment.

## Discussion

CAD is a chronic disease with a great variety of clinical courses reaching from long, stable periods to rapid progression with plaque instability and resulting acute coronary syndrome (6). Currently, the prediction of plaque development is a major challenge in clinical routine. Focusing solely on the degree of stenosis is insufficient in most patients as ≈65% of heart attacks result from the rupture of vulnerable plaques of hemodynamically insignificant subclinical changes of the vascular wall (3). Although the metabolic effects of a lipid-lowering medication can be measured easily, blood tests may not reflect changes in plaque burden and plaque composition adequately. While stress tests are important tools for the assessment of the hemodynamic relevance of coronary artery stenoses, they cannot provide any information about plaque development unless a lesion causes detectable ischemia. Coronary CTA allows for the assessment of the entire coronary tree featuring a good agreement with IVUS in the determination of the plaque burden (13, 14). Thus, changes in coronary plaques can be assessed non-invasively.

The quality of the CT scanner hardware and the reconstruction methods is of paramount importance for plaque analysis in coronary CTA. The theoretical spatial resolution of third-generation DSCT is approximately 300 μm, whereas depending on the reconstruction algorithm 415–625 μm are achieved in clinical routine (15). However, the histologically measured diameter of a fibrous cap is <65 μm (16). Therefore, it can be assumed that even with state-of-the-art equipment we can currently only see the tip of the iceberg and have to rely on image features indicating plaque instability (5, 17). While Radiomics-based approaches bear the potential to improve the assessment of advanced atheromatous lesions (18), they are still objects of research and not implemented in clinical routine.

The sole use of the Agatston score for CAD evaluation and follow-up examinations—a widely recognized, highly potent, independent predictor for a cardiovascular outcome without the need for contrast medium application (19)—is problematic as it reflects only about 20% of the actual plaque burden (20). Additionally, it remains unknown, whether an increasing Agatston score represents a “healing” of a non-calcified plaque or a progression of the atherosclerotic disease (21).

Our study showed that changes in plaque size can develop within a short time period reflecting the dynamic nature of CAD even in clinically stable patients. Within ≈2 years, 42% of the patients showed a change in the plaque burden. Regression of plaques was predominantly found in women, whereas progression was mainly observed in men, which is in line with previous studies (22, 23). While no coronaries with plaque regression at follow-up coronary CTA examinations were found in patients under statin medication in the PARADIGM study (21), previous studies reported a decrease of the CTA-determined plaque burden in patients under lipid-lowering therapy (24–26). In our study, plaque regression was only observed in patients with relatively mild CAD, implying the benefit of early statin therapy. Non-calcified and mixed plaques are potentially reversible, can rupture, completely calcify, or even regress. They may regress under anti-inflammatory medication or statin therapy or even spontaneously (24–28). Thus, the picture attained in our real-world population is quite heterogenous: On the one hand, spontaneous regression of plaques could be observed even in patients without lipid-lowering therapy. On the other hand, patients showed a progression of atherosclerosis despite medical therapy. These findings underline the necessity of sufficient and individual follow-up care in CAD patients. Currently, follow-up examinations do not include anatomical imaging mainly due to its lack of ischemia detection (6). However, the plaque burden was shown to be a strong risk factor for cardiovascular events (17, 29, 30). In addition, the morphological information on plaque composition derived from coronary CTA has a high prognostic value (5, 17). Thus, the alterations of plaque burden and composition over time as well as under medical therapy may be of great clinical interest. Motoyama showed, that patients with progression of high-risk plaques in coronary CTA had a 26.7% likelihood of cardiovascular events whereas patients without plaque progression and without high-risk plaques suffered almost no acute coronary syndrome (0.3%) (31). The SCOT-HEART study demonstrated that diagnostic coronary CTA alone significantly improves the prognosis of CAD patients over 5 years (32). This may be attributed to the correct diagnosis of CAD leading to an appropriate treatment inclusive of preventive therapies as well as the higher motivation of patients to implement healthy lifestyle modifications. Interestingly, the demonstration of the CT scan and the knowledge of the calcium score was shown to increase the compliance of CAD patients (33). Thus, coronary CTA offers the potential for an individualized assessment of the CAD progression and the effectiveness of the therapeutic regimen. Furthermore, it can simultaneously enhance the therapy adherence of the patients by visualizing plaque alterations.

In contrast to our study, most previous trials on plaque development using coronary CTA were controlled or interventional studies (e.g., assessing the impact of statins) and, thus, may not picture the clinical reality comprehensively. In our study population, a progression of plaques was observed in 36% of the study population in the follow-up CTA examination leading to a change in the clinical management in 93% of these patients (group II). The clinical management was adjusted in 66% of the entire study population demonstrating the high potential of coronary CTA examinations for therapy evaluation and optimization in CAD patients. Hence, coronary CTA is expected to be increasingly utilized to track CAD progression (34). In our opinion, prospective studies are needed to further assess the impact of follow-up CTA examinations on clinical management as well as to identify the optimal timing and patient selection criteria.

Several limitations of this study have to be considered. The study population was retrospectively included and not randomized since follow-up examinations were indicated by the referring physicians. Thus, an inclusion bias could not be excluded and confounding factors as therapy adherence were not controlled. The study population was relatively small hampering statistical analyses. Therefore, we decided to present the results descriptively. Bypassed coronary arteries or stented vessel segments were excluded from the analyses. Nevertheless, it cannot be ruled out that indirect influences, e.g., collaterals with altered hemodynamics or wall shear stress, may also play a role in the development of atherosclerosis of the non-treated vascular provinces. Lastly, dedicated software for the assessment of plaque composition and 3D plaque volume quantification was not available in this study.

## Conclusions

In conclusion, coronary CTA renders the assessment of dynamic plaque development and, thus, the evaluation of the therapeutic effectiveness in patients with chronic CAD possible. As a relevant proportion of the study population showed a plaque progression, which occurred within a relatively short time period, coronary CTA had an important impact on clinical management. Further studies must clarify, whether an individualized, targeted therapy based on follow-up CTA examinations results in prognostic benefits in CAD patients.

## Data Availability Statement

The data sets analyzed in this study are available on request from the corresponding author. They are not publicly available due data protection regulations.

## Ethics Statement

The studies involving human participants were reviewed and approved by Ethikkommission der Medizinischen Fakultät Heidelberg. Written informed consent for participation was not required for this study in accordance with the national legislation and the institutional requirements.

## Author Contributions

JG and SJB designed the study. JG, SJB, and PF performed the image analyses. FA, PF, DL, SB, JG, and SJB performed the data analyses. SS and ME curated the data. HG wrote the manuscript draft. MB, FG, RS, AS, JG, and FA revised mainly the manuscript. All authors contributed to manuscript revision, read, and approved the submitted version.

## Conflict of Interest

The authors declare that the research was conducted in the absence of any commercial or financial relationships that could be construed as a potential conflict of interest.

## Publisher's Note

All claims expressed in this article are solely those of the authors and do not necessarily represent those of their affiliated organizations, or those of the publisher, the editors and the reviewers. Any product that may be evaluated in this article, or claim that may be made by its manufacturer, is not guaranteed or endorsed by the publisher.

## References

[1] Organization WH. Global Health Estimates 2016: Deaths by Cause, Age, Sex, by Country and by Region, 2000–2016. Geneva: World Health Organization (2018).

[2] Hong YM. Atherosclerotic cardiovascular disease beginning in childhood. Korean Circ J. (2010) 40:1–9. 10.4070/kcj.2010.40.1.1 20111646 PMC2812791

[3] Chang HJ Lin FY Lee SE Andreini D Bax J Cademartiri F. Coronary atherosclerotic precursors of acute coronary syndromes. J Am Coll Cardiol. (2018) 71:2511–22. 10.1016/j.jacc.2018.02.079 29852975 PMC6020028

[4] Goldstein JA. Coronary CT Angiography: identification of patients and plaques “At Risk”. J Am Coll Cardiol. (2018) 71:2523–6. 10.1016/j.jacc.2018.02.080 29852976

[5] Williams MC Moss AJ Dweck M Adamson PD Alam S Hunter A. Coronary artery plaque characteristics associated with adverse outcomes in the SCOT-HEART study. J Am Coll Cardiol. (2019) 73:291–301. 10.1016/j.jacc.2018.10.066 30678759 PMC6342893

[6] Knuuti J Wijns W Saraste A Capodanno D Barbato E Funck-Brentano C. 2019 ESC Guidelines for the diagnosis and management of chronic coronary syndromes. Eur Heart J. (2020) 41:407–77. 10.1093/eurheartj/ehz425 31504439

[7] Leipsic J Abbara S Achenbach S Cury R Earls JP Mancini GJ. SCCT guidelines for the interpretation and reporting of coronary CT angiography: a report of the Society of Cardiovascular Computed Tomography Guidelines Committee. J Cardiovasc Comput Tomogr. (2014) 8:342–58. 10.1016/j.jcct.2014.07.003 25301040

[8] Cury RC Abbara S Achenbach S Agatston A Berman DS Budoff MJ. Coronary Artery Disease - Reporting and Data System (CAD-RADS): an expert consensus document of SCCT, ACR and NASCI: endorsed by the ACC. JACC Cardiovasc Imaging. (2019) 1099–113. 10.1016/j.jcmg.2016.05.005 27609151

[9] Kolossvary M Szilveszter B Merkely B Maurovich-Horvat P. Plaque imaging with CT-a comprehensive review on coronary CT angiography based risk assessment. Cardiovasc Diagn Ther. (2017) 7:489–506. 10.21037/cdt.2016.11.06 29255692 PMC5716945

[10] Puchner SB Liu T Mayrhofer T Truong QA Lee H Fleg JL. High-risk plaque detected on coronary CT angiography predicts acute coronary syndromes independent of significant stenosis in acute chest pain: results from the ROMICAT-II trial. J Am Coll Cardiol. (2014) 64:684–92. 10.1016/j.jacc.2014.05.039 25125300 PMC4135448

[11] Rutsch M Akin I Borggrefe M Renker M Lossnitzer D Baumann S. Coronary computed tomography-derived fractional flow reserve assessment-a gatekeeper in intermediate stenoses. Am J Cardiol. (2018) 121:778–9. 10.1016/j.amjcard.2017.12.016 29361287

[12] Wang R Renker M Schoepf UJ Wichmann JL Fuller SR Rier JD. Diagnostic value of quantitative stenosis predictors with coronary CT angiography compared to invasive fractional flow reserve. Eur J Radiol. (2015) 84:1509–15. 10.1016/j.ejrad.2015.05.010 26022519

[13] Fischer C Hulten E Belur P Smith R Voros S Villines TC. Coronary CT angiography versus intravascular ultrasound for estimation of coronary stenosis and atherosclerotic plaque burden: a meta-analysis. J Cardiovasc Comput Tomogr. (2013) 7:256–66. 10.1016/j.jcct.2013.08.006 24148779

[14] Voros S Rinehart S Qian Z Joshi P Vazquez G Fischer C. Coronary atherosclerosis imaging by coronary CT angiography: current status, correlation with intravascular interrogation and meta-analysis. JACC Cardiovasc Imaging. (2011) 4:537–48. 10.1016/j.jcmg.2011.03.006 21565743

[15] Faby S. Personal Communication. In: Görich J editor. Forchheim. Siemens Healthineers (2019).

[16] Kolodgie FD Burke AP Farb A Gold HK Yuan J Narula J. The thin-cap fibroatheroma: a type of vulnerable plaque: the major precursor lesion to acute coronary syndromes. Curr Opin Cardiol. (2001) 16:285–92. 10.1097/00001573-200109000-00006 11584167

[17] Lee JM Choi KH Koo BK Park J Kim J Hwang D. Prognostic implications of plaque characteristics and stenosis severity in patients with coronary artery disease. J Am College Cardiol. (2019) 73:2413–24. 10.1016/j.jacc.2019.02.060 31097161

[18] Kolossvary M Karady J Kikuchi Y Ivanov A Schlett CL Lu MT. Radiomics versus visual and histogram-based assessment to identify atheromatous lesions at coronary CT angiography: an *ex vivo* study. Radiology. (2019) 293:89–96. 10.1148/radiol.2019190407 31385755 PMC6776230

[19] Sakamoto A Virmani R Finn AV. Coronary artery calcification: recent developments in our understanding of its pathologic and clinical significance. Curr Opin Cardiol. (2018) 33:645–52. 10.1097/HCO.0000000000000558 30307412

[20] Rumberger JA Simons DB Fitzpatrick LA Sheedy PF Schwartz RS. Coronary artery calcium area by electron-beam computed tomography and coronary atherosclerotic plaque area. A histopathologic correlative study. Circulation. (1995) 92:2157–62. 10.1161/01.CIR.92.8.2157 7554196

[21] Lee SE Chang HJ Sung JM Park HB Heo R Rizvi A. Effects of statins on coronary atherosclerotic plaques: the PARADIGM study. JACC Cardiovasc Imaging. (2018) 11:1475–84. 10.1016/j.jcmg.2018.04.015 29909109

[22] Gu H Gao Y Wang H Hou Z Han L Wang X. Sex differences in coronary atherosclerosis progression and major adverse cardiac events in patients with suspected coronary artery disease. J Cardiovasc Comput Tomogr. (2017) 11:367–72. 10.1016/j.jcct.2017.07.002 28754436

[23] Stegman B Shao M Nicholls SJ Elshazly M Cho L King P. Coronary atheroma progression rates in men and women following high-intensity statin therapy: a pooled analysis of REVERSAL, ASTEROID and SATURN. Atherosclerosis. (2016) 254:78–84. 10.1016/j.atherosclerosis.2016.09.059 27710808

[24] Burgstahler C Reimann A Beck T Kuettner A Baumann D Heuschmid M. Influence of a lipid-lowering therapy on calcified and noncalcified coronary plaques monitored by multislice detector computed tomography: results of the New Age II Pilot Study. Invest Radiol. (2007) 42:189–95. 10.1097/01.rli.0000254408.96355.85 17287649

[25] Lo J Lu MT Ihenachor EJ Wei J Looby SE Fitch KV. Effects of statin therapy on coronary artery plaque volume and high-risk plaque morphology in HIV-infected patients with subclinical atherosclerosis: a randomised, double-blind, placebo-controlled trial. Lancet HIV. (2015) 2:e52–63. 10.1016/S2352-3018(14)00032-0 26424461 PMC4820828

[26] Zeb I Li D Nasir K Malpeso J Batool A Flores F. Effect of statin treatment on coronary plaque progression - a serial coronary CT angiography study. Atherosclerosis. (2013) 231:198–204. 10.1016/j.atherosclerosis.2013.08.019 24267226

[27] Drakopoulou M Toutouzas K Michelongona A Tousoulis D. Statins and vulnerable plaque. Curr Pharm Des. (2017) 23:7069–85. 10.2174/1381612823666171019161609 29065824

[28] Ridker PM Everett BM Thuren T MacFadyen JG Chang WH Ballantyne C. Antiinflammatory therapy with canakinumab for atherosclerotic disease. N Engl J Med. (2017) 377:1119–31. 10.1056/NEJMoa1707914 28845751

[29] Bittencourt MS Hulten E Ghoshhajra B O'Leary D Christman MP Montana P. Prognostic value of nonobstructive and obstructive coronary artery disease detected by coronary computed tomography angiography to identify cardiovascular events. Circul Cardiovascul Imaging. (2014) 7:282–91. 10.1161/CIRCIMAGING.113.001047 24550435

[30] Dey D Achenbach S Schuhbaeck A Pflederer T Nakazato R Slomka PJ. Comparison of quantitative atherosclerotic plaque burden from coronary CT angiography in patients with first acute coronary syndrome and stable coronary artery disease. J Cardiovasc Comput Tomogr. (2014) 8:368–74. 10.1016/j.jcct.2014.07.007 25301042

[31] Motoyama S Ito H Sarai M Kondo T Kawai H Nagahara Y. Plaque characterization by coronary computed tomography angiography and the likelihood of acute coronary events in mid-term follow-up. J Am College Cardiol. (2015) 66:337–46. 10.1016/j.jacc.2015.05.069 26205589

[32] Investigators S-H Newby DE Adamson PD Berry C Boon NA Dweck MR. Coronary CT angiography and 5-year risk of myocardial infarction. N Engl J Med. (2018) 379:924–33. 10.1056/NEJMoa1805971 30145934

[33] Kalia NK Cespedes L Youssef G Li D Budoff MJ. Motivational effects of coronary artery calcium scores on statin adherence and weight loss. Coronary Artery Dis. (2015) 26:225–30. 10.1097/MCA.0000000000000207 25514570

[34] Dahal S Budoff MJ. Implications of serial coronary computed tomography angiography in the evaluation of coronary plaque progression. Curr Opin Lipidol. (2019) 30:446–51. 10.1097/MOL.0000000000000645 31592788

