# Supplementary material for: Short-Time Changes in Coronary Artery Plaques Assessed by Follow-Up Coronary CT Angiography—Characteristics and Impact on Patient Management
Source: Front Cardiovasc Med. 2021 Aug 9;8:691665. doi: 10.3389/fcvm.2021.691665 (PMC8380958; doi:10.3389/fcvm.2021.691665)
Supplement: Supplementary file 1 [file Image_1.pdf]

Supplementary Material

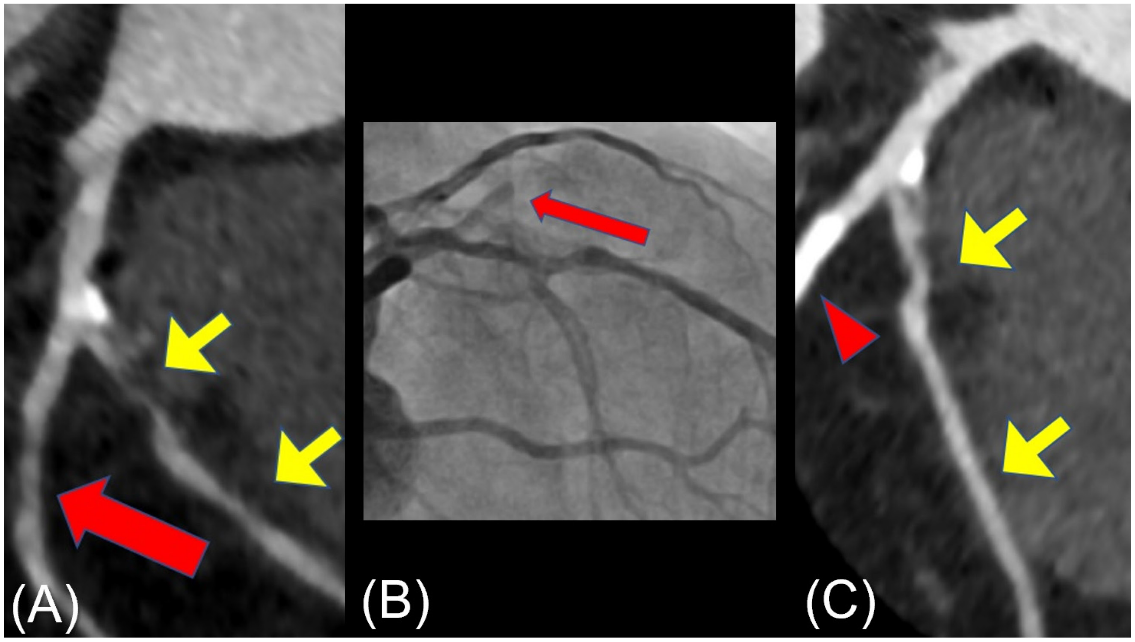

Supplementary Figure 1. Undetermined Course of CAD.

One patient was excluded from further analyses since he showed an undetermined course of the CAD and, thus, could not be assigned to any group. **(A)** At the initial presentation, a moderate proximal LAD stenosis (yellow arrows) and a non-calcified plaque with mild narrowing of the first diagonal ramus (red arrow) were found with hemodynamic significance (CT_{FFR} 0.60). **(B)** One week after the examination but before the revascularization, the patient suffered a myocardial infarction with the closure of the first diagonal ramus as shown in the coronary angiography (red arrow). **(C)** After 11 months, the follow-up coronary CTA showed an open coronary artery stent (arrowhead) and a spontaneous regression of the non-calcified plaque of approximately 50% of the LAD (yellow arrows), although statin therapy was rejected. Yet, a mild progression of the non-calcified plaques in the distal LAD could be observed.

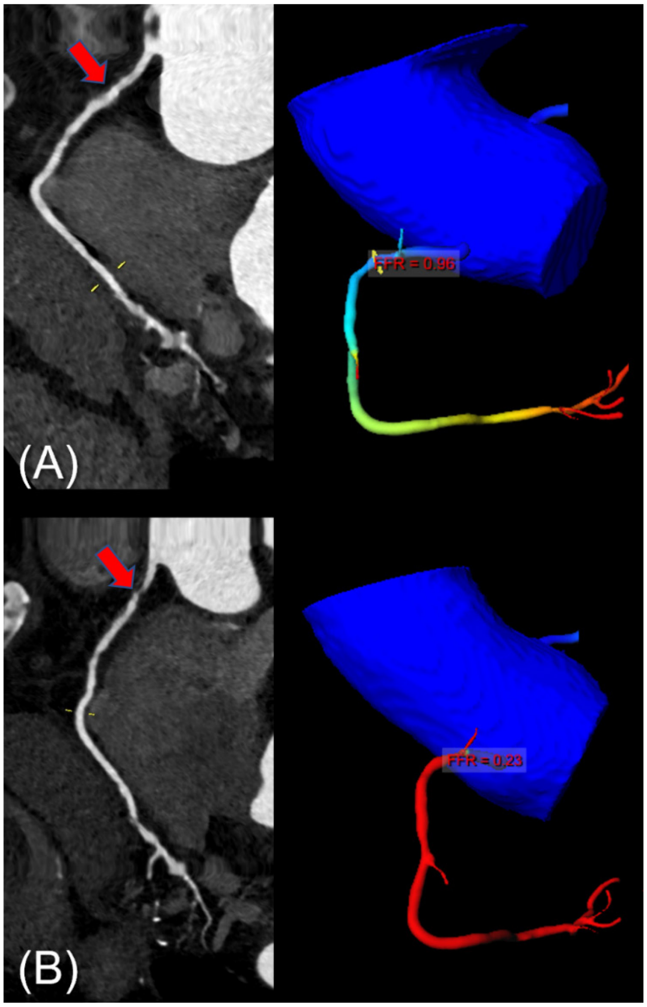

Supplementary Figure 2. Example of CT_{FFR}-confirmed non-calcified plaque progression.

(A) The baseline coronary CTA showed a perivascular inflammation (arrow) around the proximal RCA with a mild stenosis without hemodynamic relevance in the CT_{FFR} evaluation. The patient had significant stenoses of the LAD und LCX and underwent coronary bypass surgery (not shown). (B) The follow-up coronary CTA after 14 months, which was triggered by increasing angina, revealed a significant increase in the inflammation of the RCA resulting in a high-grade stenosis with a considerably reduced CT_{FFR} of 0.23. Of note, CT_{FFR} values of the LAD were not reliable due to the open bypass graft.
